# Co-occurrence of Alpha-1 Antitrypsin Deficiency (AATD) and Common Variable Immunodeficiency (CVID): A Case Report

**DOI:** 10.7759/cureus.96755

**Published:** 2025-11-13

**Authors:** Aysun Aynaci, Fatma Merve Tepetam, Erdogan Cetinkaya, Nihal Yildirim, Şeyma Özden

**Affiliations:** 1 Allergy and Immunology, Süreyyapaşa Chest Diseases and Thoracic Surgery Training and Research Hospital, Istanbul, TUR; 2 Chest Diseases, Yedikule Chest Diseases and Thoracic Surgery Training and Research Hospital, Istanbul, TUR

**Keywords:** alpha-1 antitrypsin deficiency, bronchiectasis, common variable immunodeficiency (cvid), hypogammaglobulinemia, immunologic replacement therapy

## Abstract

Alpha-1 antitrypsin deficiency (AATD) and common variable immunodeficiency (CVID) are distinct disorders, and their co-occurrence is rare. We report a 47-year-old man with a history of smoking and chronic obstructive pulmonary disease (COPD), diagnosed with the PI*MZ genotype of AATD and treated with human alpha1-proteinase inhibitor (PROLASTIN-C®, Grifols Therapeutics, Clayton, NC, USA). Despite therapy, he experienced recurrent respiratory infections and pneumonia. Further evaluation revealed hypogammaglobulinemia and immune dysregulation consistent with CVID, leading to bronchiectasis. Combined treatment with alpha-1 antitrypsin (AAT) replacement and intravenous immunoglobulin (IVIG) was initiated, emphasizing the importance of comprehensive genetic and immunologic evaluation and tailored therapy in complex respiratory cases. Further studies are needed to clarify the relationship between AATD and CVID and optimize management strategies.

## Introduction

Alpha-1 antitrypsin (AAT) is a protein that belongs to the serine protease inhibitor family and is primarily produced by hepatocytes. Additionally, it is produced in smaller amounts by monocyte-derived macrophages and dendritic cells, alveolar macrophages, pancreatic cells, enterocytes and endothelial cells, activated neutrophils, and some cancer cells [1]. In addition to its role as an antiprotease, AAT exhibits other biological effects, such as its ability to regulate inflammation and apoptosis [2]. Alpha-1 antitrypsin deficiency (AATD) is an inherited genetic disorder transmitted in an autosomal codominant fashion. Its prevalence is estimated at approximately one in 2,700 individuals in Northern Europe and about one in 18,000 in Central Europe [3]. AATD significantly increases the risk of developing chronic obstructive pulmonary disease (COPD), emphysema, and chronic bronchitis, as well as liver cirrhosis, panniculitis, and cytoplasmic anti-neutrophil cytoplasmic autoantibody (c-ANCA)-positive vasculitis. These complications arise due to an imbalance in protease-antiprotease activity and the toxic effects of AAT polymer accumulation [4]. The condition is caused by pathogenic variants in the SERPINA1 gene, located on chromosome 14q32.13. While the common M allele corresponds to normal circulating AAT levels, the S and Z alleles are linked to significantly reduced serum concentrations, approximately 60% and 15% of normal levels, respectively. Around 95% of clinically diagnosed AATD cases involve individuals who are homozygous for the Z allele (PI*ZZ genotype), while the remaining cases typically involve compound heterozygosity such as PI*SZ, PI*MZ, or combinations of rare deleterious variants [5].

Predominantly antibody deficiencies (PADs) constitute the most common form of inborn errors of immunity (IEIs) in humans. These disorders exhibit considerable heterogeneity in clinical presentation, age of onset, and disease progression. Among the PADs, common variable immunodeficiency (CVID) is the most frequently diagnosed subtype. CVID is marked by persistent hypogammaglobulinemia affecting all major immunoglobulin (Ig) isotypes, a lack of isohemagglutinins, and inadequate immune responses to vaccines. Clinically, it manifests with recurrent infections, autoimmunity, benign or malignant lymphoproliferation, and granulomatous inflammation.

Although some studies have explored the role of AAT and AATD in influencing PAD phenotypes, the available data remain limited and inconclusive. Furthermore, the underlying genetic causes of CVID are still unknown in the majority of cases. To date, only 14 monogenic forms of CVID have been identified and are listed in the Online Mendelian Inheritance in Man (OMIM) database.

In the first study by Sansom et al. [6], a trend towards an association of the Z allele of AAT was observed in a subgroup of CVID patients with bronchiectasis. In the latest study in which detailed SERPINA genetic analyses were performed, the presence of the Z allele was significantly associated with liver disease [2].

This case presents a patient diagnosed with the MZ phenotype of AATD without liver involvement, who experienced frequent hospitalizations due to recurrent bronchiectasis-related pneumonia. Further investigations led to a diagnosis of CVID.

## Case presentation

A 47-year-old male patient has been followed and treated for COPD for five years. The patient, who had a history of smoking one pack of cigarettes per day for 33 years, quitted smoking one month ago, and he was working as a sales consultant. 

In 2023, the patient was diagnosed with AATD at an external center, with genetic testing revealing an M/Z allele deficiency (PI*MZ). The AAT level was measured as 67 mg/dL (90-200 mg/dL). This qualitative test for AATD genotyping identifies the 14 most common allele variants simultaneously. Consequently, the patient was started on human alpha1-proteinase inhibitor (PROLASTIN-C®, Grifols Therapeutics, Clayton, NC, USA) replacement therapy for AATD. He experienced 15-20 respiratory infections annually, primarily bacterial lower respiratory tract infections, with one episode of pneumonia in the last year. Over the past year, the patient had five hospitalizations due to acute lower respiratory tract infections and COPD exacerbations. Therefore, he was referred to our immunology and allergy clinic to be evaluated for immunodeficiency.

**Figure 1:**
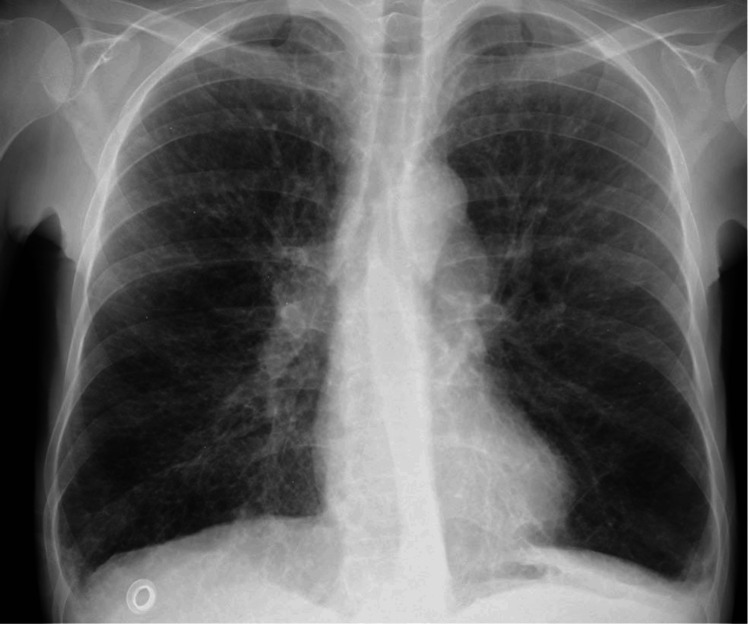
PA chest X-ray showing the diaphragm contours were flattened and there were an increase in bilateral bronchovascular scarring and ventilation in the basal areas. The PA chest radiograph shows bilateral diaphragmatic flattening, increased bronchovascular markings suggestive of bronchial wall thickening or scarring, and signs of hyperinflation with enhanced basal ventilation. PA: posterior-anterior

**Figure 2:**
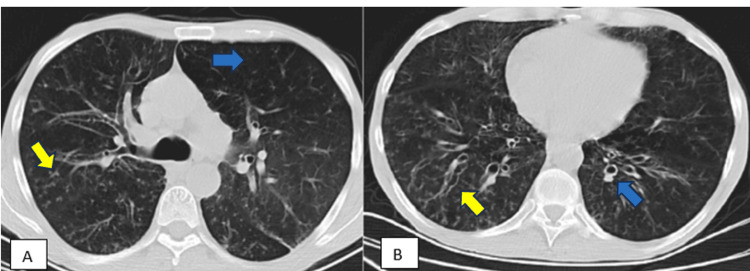
Thoracic CT showing widespread central and peripheral nodular infiltrations, moderate bronchiectasis, air-trapping areas, and bronchial wall thickening in both lungs, predominantly in the middle and lower zones. (A) CT of the thorax shows widespread peripheral and central nodular infiltrations (yellow arrow) and air-trapping areas (blue arrow). (B) CT of the thorax shows moderate bronchiectasis (yellow arrow) and bronchial wall thickening in both lungs (blue arrow), predominantly in the middle and lower zones.

The patient had no family history of immunodeficiency. There were no consanguineous marriage and no siblings who died at an early age in the family. The patient was the only child of the family. In the physical examination, auscultation revealed the presence of expiratory rhonchi and bibasilar crackles. In the posterior-anterior (PA) chest radiograph dated April 2024 (date of referral to the allergy clinic), the diaphragm contours were flattened, and there were an increase in bilateral bronchovascular scarring and ventilation in the basal areas (Figure 1). At the same time, a thoracic CT scan revealed widespread central and peripheral nodular infiltrations, moderate bronchiectasis, areas of air trapping, and bronchial wall thickening in both lungs, predominantly in the middle and lower zones. Atelectatic changes, poorly ventilated peripheral parenchymal areas, focal areas of increased aeration, and ground-glass opacities were observed. Bilateral segmental and subsegmental peribronchial thickening was noted. There were areas of thickening and retraction on the bilateral costal pleural surfaces (Figure 2). 

In laboratory tests, complete blood count revealed lymphopenia (1.10×10^9^/L) and normal sedimentation. The C-reactive protein (CRP) test was slightly high (6.12 mg/dL). In patients with lymphopenia and recurrent infections, evaluation of Ig levels and specific antibody responses is recommended to assess for a preliminary diagnosis of CVID. Antibody levels are reported as follows, and the results were confirmed by repeating the tests. Low IgG and subgroups and IgA are revealed (Table 1). In CVID, immune dysfunction is not limited to infections; autoimmunity is also common. Therefore, autoantibody testing is performed to identify possible coexisting autoimmune diseases. All autoimmune markers were negative in the patient (anti-nuclear antibody (ANA): negative; ANCA: negative; anti-myeloperoxidase (anti-MPO): <2 U/mL (0-20 U/mL); anti-proteinase 3 (anti-PR3): <2 RU/mL (0-20 RU/mL)). Lıver functıon tests were also normal (aspartate aminotransferase (AST): 20 U/L (0-40 U/L); alanine aminotransferase (ALT): 23 U/L (0-41 U/L)). *Aspergillus fumigatus*-specific IgE, mold mix-specific IgE, and sputum fungal culture were negative. Vaccination responses for rubella, toxoplasmosis, cytomegalovirus (CMV), hepatitis A virus (HAV), and hepatitis B virus (HBV) were negative. No variants were found in the immunodysregulation genetic panel.

**Table 1:** First-line laboratory test results. CRP: C-reactive protein; ANA: anti-nuclear antibody; ANCA: anti-neutrophil cytoplasmic autoantibody; anti-MPO: anti-myeloperoxidase; anti-PR3: anti-proteinase 3; AST: aspartate aminotransferase; ALT: alanine aminotransferase; IgG: immunoglobulin G

	Test result	Reference value
Lymphocyte	1.10×10^9^/L	0.6-3.4×10^9^/L
Sedimentation	6 mm	5-20 mm
CRP	6.12 mg/dL	0-5 mg/dL
ANA	Negative	Negative (<1/100)
ANCA	Negative	Negative (<1/10)
Anti-MPO	<2 U/mL	0-20 U/mL
Anti-PR3	<2 RU/mL	0-20 RU/mL
AST	20 U/L	0-40 U/L
ALT	23 U/L	0-41 U/L
IgG	0.75 g/L	7-16 g/L
IgM	0.77 g/L	0.4-2.3 g/L
IgA	<0.21 g/L	0.7-4 g/L
IgG1	0.96 g/L	4.05-10.11 g/L
IgG2	0.41 g/L	1.69-7.86 g/L
IgG3	0.003 g/L	0.11-0.85 g/L
IgG4	0.004 g/L	0.03-2.01 g/L
Total IgE	0.2 IU/mL	0-100 IU/mL

Respiratory function tests of the patient before and after human alpha1-proteinase inhibitor replacement therapy were as follows: forced expiratory volume in one second (FEV1): 1.14 L (31%), forced vital capacity (FVC): 1.67 L (34.80%), and FEV1/FVC: 59.88% and FEV1: 1.28 L (34.59%), FVC: 2.48 L (52.76%), and FEV1/FVC: 51.61%.

After the human alpha1-proteinase inhibitor (PROLASTIN-C®) replacement therapy was given to the patient, pulmonary function test results showed improvement, and clinically significant improvement was also observed.

When lymphocyte subgroups were examined, CD19+, CD20+, CD4/CD8 ratio, and switched memory B cells (CD27+ IgM- IgD- cells in peripheral B lymphocytes) were found to be low. The results of flow cytometric analysis are given in Table 2.

**Table 2:** Flow cytometric analysis of lymphocyte subgroups.

	Test result	Reference value
CD3+	88.79%	62-88%
CD4+	24.58%	35.3-61.1%
CD8+	61.5%	11.2-37.3%
CD4/CD8 ratio	0.39	1-3
CD19+	1.08%	6.3-20%
CD20+	1.09%	7.1-23.8%
Switched memory B cells	5%	9.2-18.9%
CD16+/CD56+	9.3%	3.2-23.7%

The patient was diagnosed with immunodeficiency and started on intravenous immunoglobulin (IVIG) 0.5 g/kg (30 g) replacement therapy. He had a significant reduction in severe respiratory tract infections after IVIG replacement therapy and had no attacks of pneumonia.

## Discussion

SERPINA genotyping was performed in our case, a 47-year-old male patient with a 33-pack-year smoking history, due to advanced COPD, and the PI*MZ genotype was detected in this patient, although there was no significant emphysema in lung radiology and the AAT level was normal. We presented a case who was referred to our immunology and allergy clinic to be evaluated for primary immunodeficiency (PID) disease due to the presence of air-trapping areas and bronchiectatic changes on thoracic CT and a history of recurrent upper respiratory tract infections and attacks of pneumonia and who was receiving human alpha1-proteinase inhibitor (PROLASTIN-C®) replacement therapy for one year. In investigations of the patient, besides lymphopenia, IgG and its subgroups and IgA were low, and the CD4/CD8 ratio was reversed. As the CD19+ and CD20+ B-cell count was greater than 1%, agammaglobulinemia was excluded. However, switched memory B cells were reduced, vaccine responses were negative, and the findings were consistent with a diagnosis of CVID. In the limited number of studies evaluating AATD in CVID patients, the most frequently detected phenotype is the MZ phenotype, as in our case [6-8]. Consistent with studies, our case was accompanied by bronchiectasis but not by autoimmunity [2-6]. However, in our case, chronic liver disease, as associated with the PI*MZ genotype, was not detected [2]. 

The co-occurrence of AATD and PADs is extremely rare. In the limited number of studies and published cases, it has been found that PADs and CVID are the most common diseases associated with AATD, while PI*MZ is the most common genotype. According to the studies evaluating AATD, one of which included 43 CVID patients and the other 40 PADs patients (CVID: 24), the most common genotype was PI*MZ (3/43, 2/27). However, neither of these two studies found a significantly increased frequency for the occurrence of the Z allele when compared with the healthy population. But in the first study conducted by Sansom et al., when subgroups of CVID patients were analyzed for bronchiectasis, it was revealed that there was a tendency towards bronchiectasis in patients with the Z allele (all four patients had bronchiectasis) [6]. In another study that included the largest number of patients, the prevalence of the PI*MZ genotype was found to be 4.5% in PID patients (5/110) [8]. In the recent study, in which SERPINA1 genetic analyses and clinical features were detailed, 80 patients with PAD, including 70 with CVID, were examined. Ten CVID patients were found to carry heterozygous pathogenic SERPINA1 defects with normal AAT levels. The second most common defect phenotype detected in three patients was MZ, which was found to be significantly associated with the development of chronic liver disease but was found to be less likely to develop autoimmune disease [2]. Compatible with this literature, our case was a patient with the PI*MZ genotype with normal AAT levels, without autoimmune disease, diagnosed with CVID; contrary to the literature, no liver disease accompanied.

The presence of hypogammaglobulinemia and low Ig levels in AATD can be explained by several mechanisms. AAT is an important inhibitor protein that regulates the activity of proteases like neutrophil elastase. In AATD, neutrophil elastase becomes uncontrollably active, leading to a proteolytic imbalance that affects the regular functioning of the immune system [9]. High neutrophil elastase activity can cause damage to the membranes of immune cells and the extracellular matrix, preventing an effective immune response [8]. Additionally, chronic inflammation can negatively impact Ig production, leading to hypogammaglobulinemia. The deficiency of AAT can hinder the differentiation and maturation of B cells, causing Ig to remain at low levels [10]. The polymerization of AAT in liver cells and its intracellular retention negatively affect the function of these cells and weaken the immune response [10].

AAT replacement therapy is crucial in treating AATD to protect lung function. However, IVIG replacement therapy is also essential for treating CVID. This therapy regulates Ig levels, enhancing the patient's resistance to infections and ensuring the proper functioning of the immune system. Therefore, both treatment methods should be applied together. As noted by Henao and Craig [9], AAT replacement therapy increases AAT levels, reducing the damage caused by neutrophil elastase to the lung tissue, while IVIG therapy can provide benefits beyond the correction of immunodeficiency, including the modulation of underlying autoimmunity and potential protective effects in patients with associated malignancy.

## Conclusions

Our case emphasizes that genotyping should be performed in severe COPD patients with bronchiectatic changes without radiologically evident emphysema, despite normal AAT levels, and that COPD patients presenting with recurrent infections should be referred to the immunology and allergy clinic to be evaluated for PID. In addition, increasing awareness of this rare association will contribute to the early diagnosis of patients with similar symptoms and the early initiation of appropriate treatment strategies to prevent the progression of both diseases with high mortality.
